# Investigating the Consequences of eIF4E2 (4EHP) Interaction with 4E-Transporter on Its Cellular Distribution in HeLa Cells

**DOI:** 10.1371/journal.pone.0072761

**Published:** 2013-08-21

**Authors:** Dorota Kubacka, Anastasiia Kamenska, Helen Broomhead, Nicola Minshall, Edward Darzynkiewicz, Nancy Standart

**Affiliations:** 1 Department of Biochemistry, University of Cambridge, Cambridge, United Kingdom; 2 Division of Biophysics, Institute of Experimental Physics, Faculty of Physics, University of Warsaw, Warsaw, Poland; The John Curtin School of Medical Research, Australia

## Abstract

In addition to the canonical eIF4E cap-binding protein, eukaryotes have evolved sequence–related variants with distinct features, some of which have been shown to negatively regulate translation of particular mRNAs, but which remain poorly characterised. Mammalian eIF4E proteins have been divided into three classes, with class I representing the canonical cap-binding protein eIF4E1. eIF4E1 binds eIF4G to initiate translation, and other eIF4E-binding proteins such as 4E-BPs and 4E-T prevent this interaction by binding eIF4E1 with the same consensus sequence YX _4_Lϕ. We investigate here the interaction of human eIF4E2 (4EHP), a class II eIF4E protein, which binds the cap weakly, with eIF4E-transporter protein, 4E-T. We first show that ratios of eIF4E1:4E-T range from 50:1 to 15:1 in HeLa and HEK293 cells respectively, while those of eIF4E2:4E-T vary from 6:1 to 3:1. We next provide evidence that eIF4E2 binds 4E-T in the yeast two hybrid assay, as well as in pull-down assays and by recruitment to P-bodies in mammalian cells. We also show that while both eIF4E1 and eIF4E2 bind 4E-T via the canonical YX _4_Lϕ sequence, nearby downstream sequences also influence eIF4E:4E-T interactions. Indirect immunofluorescence was used to demonstrate that eIF4E2, normally homogeneously localised in the cytoplasm, does not redistribute to stress granules in arsenite-treated cells, nor to P-bodies in Actinomycin D-treated cells, in contrast to eIF4E1. Moreover, eIF4E2 shuttles through nuclei in a Crm1-dependent manner, but in an 4E-T–independent manner, also unlike eIF4E1. Altogether we conclude that while both cap-binding proteins interact with 4E-T, and can be recruited by 4E-T to P-bodies, eIF4E2 functions are likely to be distinct from those of eIF4E1, both in the cytoplasm and nucleus, further extending our understanding of mammalian class I and II cap-binding proteins.

## Introduction

Control of translation in eukaryotes is critical for proper development, cell growth and proliferation, and the most highly regulated level is at initiation. A key player in translation initiation is eIF4E, the mRNA 5′ cap-binding protein, whose aberrant expression and phosphorylation promotes tumorigenesis and which has also been implicated in mechanisms underlying senescence and autism [1–4]. Translation initiation begins with the rate-limiting binding of the eIF4F (eIF4E, eIF4G and the RNA helicase eIF4A) complex to the 5' cap and is completed upon start codon recognition by the pre-initiation complex. eIF4E recruits ribosomes to mRNA 5’ ends through specific binding to eIF4G, which also contains binding sites for eIF3, associated with the small ribosomal subunit. eIF4E sandwiches the m^7^G cap via conserved tryptophan residues and binds the consensus YXXXXLϕ sequence in eIF4G (where ϕ is hydrophobic and X is any amino acid) on its convex side. Well-characterised inhibitors of translation initiation known as eIF4E-binding proteins (4E-BPs) contain similar YX _4_Lϕ motifs. These small proteins have been described as molecular mimics of eIF4G, since they act by competing for the same binding site of eIF4E. When hypophosphorylated in quiescent cells, 4E-BPs associate with eIF4E, preventing eIF4E from binding eIF4G, and blocking the formation of the translation initiation complex [5–7].

In addition to this well-characterised canonical eIF4E cap-binding protein, eukaryotes have evolved sequence-related variants with distinct features, some of which have been shown to regulate translation of particular mRNAs rather than participate in global translation initiation. For example, *Drosophila melanogaster* possess eight eIF4E proteins, with individual developmental expression profiles, and with varying abilities to bind the cap, eIF4G and 4E-BP [8,9]. In *C. elegans*, the five eIF4E proteins differ in their recognition of mono and trimethylated caps, and regulate sub-sets of mRNAs [10,11]. Mammalian eIF4E proteins have been divided into three classes, with class I representing the canonical cap-binding protein [12,13].

Our study focuses on human eIF4E2, a 245 amino acid long ubiquitously expressed protein, also known as 4EHP (eIF4E homologous protein) or eIF4EL3 (eIF4E-like 3), a class II protein, which is 30% identical and 60% similar in sequence to eIF4E1 [13,14]. eIF4E2 does not interact with eIF4G and binds 4E-BPs relatively weakly [12,14,15]. Its affinity for the cap is approx 30-100 x lower than that of eIF4E1, largely due to an extension of one loop which creates the ligand binding site and thus negatively affects formation of the three stacked aromatic rings, Trp124/m^7^Gua/Tyr78, and also different arrangements of basic amino acids interacting with the phosphate chain [15,16]. Hence eIF4E2, on its own, will not compete with eIF4E1 for mRNA effectively, but may do so with a partner protein. Indeed its *Drosophila* homologue, d4EHP (eIF4E-8) binds Bicoid, an RNA-binding protein which recognises a 3’ UTR element in *caudal* mRNA to specifically repress its translation [17] and, similarly, mouse 4EHP binds cytoplasmic Prep1 inhibiting *Hoxb4* translation [18]. Recently, Morita et al. showed that eIF4E2, which forms a translational repressor complex with GIGYF2 (Grb10-interacting GYF protein 2) and zinc finger protein 598, is essential for mammalian development, since eIF4E2 ko mice are not viable, with the embryos dying perinatally [19].

We investigate here the interaction of eIF4E2 with 4E-T(ransporter), an eIF4E-binding protein. Mammalian 4E-T was first characterized as a large protein which binds eIF4E via YX _4_Lϕ and prevents eIF4E interacting with eIF4G [20]. Indeed, 4E-T was shown to inhibit cap-dependent translation as well as to regulate ARE-(AU-rich) mRNA stability [20]. 4E-T is a component of P-(rocessing) bodies and a nucleocytoplasmic protein which transports eIF4E into nuclei [21–23]. P-bodies, distinct cytoplasmic foci, contain mRNA, microRNAs, mRNA decay enzymes, and RNA-binding proteins/translational repressors but not ribosomes, and are understood to participate in mRNA decay and in reversible translational repression including that mediated by microRNAs. In addition to decapping, deadenylase and exonuclease activities, components include eIF4E, the only translation initiation resident in P-bodies, p54/rck RNA helicase, Pat1b, Lsm14A, as well as 4E-T [24–26].

Control of translation is of pivotal importance during early development, particularly during meiotic maturation and early embryogenesis, as transcription is shut down. Previously, we showed that 4E-T is a major component of CPEB (cytoplasmic polyadenylation element-binding protein) mRNP in *Xenopus* oocytes [27,28]. CPEB, an RNA-binding protein, regulates 3’ UTR CPE-containing mRNAs such as cyclin B mRNA by acting both as a translational repressor in the oocyte and a translational activator in eggs, where it participates in cytoplasmic polyadenylation [29,30]. During the course of *Xenopus* oogenesis (stage I-VI), the class I eIF4E cap-binding proteins are differentially expressed, with the early expressed eIF4E1b protein being replaced by the closely related canonical eIF4E1 protein. We also showed that CPEB RNP obtained by immunoprecipitation and gel filtration contained Xp54 RNA helicase, Pat1a, Lsm14B, 4E-T and eIF4E1b as the most abundant protein partners of CPEB, resembling thus P-bodies. All these factors are expressed in early stages of oogenesis, and form a very stable complex [27]. While the detailed function of eIF4E1b in CPEB RNP remains to be established, it is interesting to note that this protein variant, which binds m^7^GTP-Sepharose inefficiently [27], is evolutionarily conserved, arising in Tetrapoda as a result of the ancestral eIF4E locus duplication. Moreover, EST evidence suggests its oocyte-restricted expression is also conserved [31]. We proposed a repressed closed loop model whereby CPEB bound to regulated mRNA via their 3’ UTR CPE elements interact directly or indirectly with 4E-T which binds eIF4E1b, precluding eIF4G recognition of the cap structure, and thereby inhibiting translation [27,32]. *Drosophila* Cup, partially related in sequence to vertebrate 4E–T, has similarly been proposed to inhibit *oskar* and *nanos* mRNAs by interacting with the 3’ UTR-binding proteins Bruno and Smaug respectively, as well as eIF4E [33,34].

In view of our interest in cap-binding proteins interactions with 4E-T, we first assessed their expression in HeLa and HEK293 cells, and show that eIF4E:4E-T ratios range from 3:1 to 50:1, depending on the cell line and the cap-binding protein. We demonstrate using several lines of evidence that eIF4E2 binds 4E-T, and that while both eIF4E2 and eIF4E1 bind 4E-T via the YX _4_Lϕ motif, nearby downstream sequences also influence eIF4E1/2:4E-T interactions. eIF4E2, normally homogeneously localised in the cytoplasm, as is eIF4E1, however does not redistribute to stress granules in arsenite-treated cells, nor to P-bodies in Actinomycin D-treated cells, in contrast to eIF4E1. Furthermore, eIF4E2 shuttles through nuclei in a Crm1-dependent manner, but in an 4E-T–independent manner, also unlike eIF4E1. Altogether we conclude that while both cap-binding proteins interact with 4E-T, and can be recruited by 4E-T to P-bodies, eIF4E2 nuclear and cytoplasmic functions are likely to be distinct from those of eIF4E1.

## Materials and Methods

### Plasmids and protein expression in E. coli

Human eIF4E2 cDNA, amplified from pcDNA3-HA-eIF4E2 vector (gift from Nahum Sonenberg) by PCR was subcloned into pEGFP-C1 (EcoRI/XmaI restriction sites) and yeast vectors, pGADT7 and pGBKT7 (BD Biosciences-Clontech), using NdeI/BamHI sites. Similarly, human 4E–T cDNA in the pEYFP-C1 vector (gift from Rheinhard Luhrmann [22]) was subcloned into pEGFP-C1 (KpnI/XmaI) and yeast vectors (NdeI/BamHI). eIF4E2 cDNA C-terminally truncated by 69 bp was amplified by PCR and recloned into pcDNA3-HA vector (BamHI/XhoI). FLAG-MS2-4E-T was constructed by inserting 3xFLAG epitope DNA into the NcoI site of the MS2-4E-T plasmid [27] using Pfu DNA polymerase (Stratagene), and similarly for the control FLAG-MS2 control construct. Mutagenesis of 4E-T was carried out in a two step process. For the human protein, Y30A and Y55A were mutated first, followed by LL35/36 AA and VW60/61 AA respectively, and similarly in the case of *Xenopus* 4E-T (Y28,Y53, LL33/34 AA, VW58/59 AA respectively). All cloning and mutagenesis, with the indicated oligonucleotides (Table S1), was carried out using Stratagene QuikChange site-directed mutagenesis kit and verified by sequencing.

Recombinant His-4E-T was prepared by the Novagen autoinduction system and denaturing purification on NTA-agarose in 8M urea and elution with pH 4.5 buffer, according to Qiagen. Recombinant eIF4E1 and eIF4E2 proteins were expressed as inclusion bodies in Rosetta2 (DE3), then purified under denaturing conditions (4-6 M guanidinium hydrochloride) and refolded by one step dialysis as described in [16]. Additional purification by anion-exchange chromatography gave homogenous fractions of proteins.

### Protein quantitation

Quantitative analysis of endogenous eIF4E1, eIF4E2 and 4E-T was performed by comparison of a concentration series of protein lysates obtained from HeLa and HEK293 cells with known amounts of purified recombinant protein counterparts loaded on the same gel. Cultures of HeLa and HEK293 cells were prepared in the same size 6-well plates and incubated in parallel. Cells from both cultures were counted using a hemocytometer, and lysates were prepared in RIPA buffer, whose protein concentration was determined by the Bradford assay. Western blots with a concentration series of recombinant eIF4E1, eIF4E2 and 4E-T proteins, quantitated with known amounts of BSA, alongside lysates from HeLa and HEK293 cells, were developed with ECL.

### Y2H double transformations and selection

A yeast two-hybrid assay was performed using the GAL4-based two-hybrid system adopted from Clontech. pGBK-protein constructs were transformed into AH109 cells on selective medium (minimal SD base supplemented with 2% glucose and the required dropout solution, -Trp in this case). Singly transformed strains were used as a yeast stock in which to transform the pGAD-protein constructs. Double transformed cells were selected on -Leu/-Trp dropout medium suitable for pGBKT7 and pGADT7 vectors containing *TRP1* and *LEU2* nutritional markers, respectively. pGBKT7 constructs were verified that they did not autonomously activate reporter genes and interaction of proteins was checked on high stringency -Ade/-His/-Leu/-Trp dropout medium. Lamin C (pGBKT7-Lamin) and SV40 large T-antigen (pGADT7-T) co-transformants were used as negative controls.

### Cell culture, transfection and drug treatments

HeLa and HEK293 cells were routinely grown in DMEM supplemented with 10% fetal calf serum. Transient transfections were performed in 24-well plates using 1 μl of Lipofectamine 2000 (Invitrogen) and 0.5 μg of plasmid constructs, or in 6-well plates using 3 μl of Lipofectamine 2000 and 3 µg of siRNA. The 4E-T siRNA was 5’ CAGUCGAGUGGAGUGUACAUUGUdTdT, purchased from Thermo Scientific, and the control β-globin siRNA was described in [35]. Cells were fixed and stained with the appropriate antibody 24 hrs after plasmid transfection or 48 hrs after siRNA transfection.

To induce oxidative stress, cells were incubated in media supplemented with 0.5 mM sodium arsenite (Sigma) for 30 min, then allowed to recover for 30 min in the absence of arsenite. For transcription inhibition of RNA polymerase I and II, 4 µM Actinomycin D (ActD) (in ethanol) was used. To inhibit Crm1-dependent export, Leptomycin B (LMB) (in methanol) (Sigma) was used at a final concentration of 10 nM for 5 hrs. The reported outcomes of these treatments were seen in all cells. Addition of the same volume of methanol or ethanol to the cells was used as a control for these inhibitor experiments. Cells were scraped in phosphate-buffered saline (PBS) and resuspended in RIPA buffer (50 mM Tris-HCl pH 8, 150 mM NaCl, 1% Triton X-100, 0.5% NaDOC, 0.1% SDS) or NET buffer with non-ionic detergent for immunoprecipitation assays (50 mM Tris-HCl pH 7.5, 150 mM NaCl, 1 mM EDTA, 0.5% Nonidet P-40, 0.25% gelatin) and incubated on ice 20 min. Buffers for lysis were supplemented with Complete Protease Inhibitor cocktail (Roche). Soluble proteins were recovered after centrifugation at 10 000 rpm at 4^°^C for 10 min and quantitated by the Bradford method.

### Immunofluorescence

Cells were plated on 13 mm glass coverslips in 24-well plates. Cells were fixed in 4% paraformaldehyde for 20 min, washed three times with phosphate buffered saline (PBS) and permeabilized in PBS with 0.5% Triton X-100 for 4 min 30 sec followed by three PBS washes. Cells were incubated with primary antibodies for 1 hr using chicken eIF4EL3/eIF4E2 (1:200; Novus Biologicals), mouse eIF4E1 (1:1000; Santa Cruz), goat 4E-T (1:200; Abcam), rabbit Pat1b (1:100), rabbit rck/p54 (1:1000; Bethyl Laboratories), rat HA (1:200; Roche), mouse G3BP (1:200; BD Biosciences), goat TIAR (1:500; Santa Cruz). The cells were then washed three times in PBS, followed by the incubation of the secondary antibodies conjugated to Rhodamine Red-X RRX, Alexa Fluor 488 or Cumarin AMCA fluorescent probes used at a 1:1000 dilution (Jackson ImmunoResearch Laboratories) for 1 hr. After rinsing three times with PBS, cells were stained with DAPI (1.25 μg/ml) for 10 sec. The coverslips were mounted in Citifluor (Citifluor Labs). All steps were performed at room temperature. Cells were observed under a Zeiss Axioimager M1 fluorescent microscope with a Plan-Apochromat 100/1.4 Oil DIC objective. Where indicated, >100 cells from several independent experiments were counted to assess the number of cells with P-bodies containing eIF4E2 and 4E-T (or as stated). We did not distinguish between small or large foci, or count the number of P-bodies per cell.

### GFP-Trap pull-down assays

GFP-Trap® Magnetic beads (Chromotek) (25 µl per sample) were re-suspended in 250 µl ice-cold NET buffer in hydrophobic tubes. Washed beads were incubated with 1 mg lysate protein, prepared from GFP-4E-T transfected HEK293-T cells, in 1 ml NET buffer supplemented with Protease Inhibitor cocktail, for 1.25 h at 4°C with constant rotation. Supernatants were then removed, beads washed and associated proteins eluted in SDS sample buffer (2% SDS, 80mM Tris-HCl pH 6.8, 10% glycerol, 5% β-mercaptoethanol, 0.0001% bromophenol blue) prior to gel electrophoresis and western blotting.

### Immunoprecipitation of eIF4E2

Chicken IgY Precipitating Resin (GenScript) consisting of agarose beads coupled to Goat Anti-Chicken IgY was used for immunoprecipitation of human eIF4E2. First, 10 µl of beads were incubated with 0.5 to 2 µl of chicken α-eIF4E2 (no antibody for control) in 1 ml NET buffer for 2 hrs at 4^°^C with constant rotation. The beads were then washed before incubation with 1 mg of protein lysate prepared from HeLa cells transfected with HA-eIF4E2 plasmid in 1 ml NET buffer supplemented with Protease Inhibitor cocktail, using the same conditions. Following extensive washing of the beads, associated proteins were eluted with 10 µl of SDS sample buffer.

### Immunoprecipitation of Xenopus 4E-T

*In vitro* transcribed mRNAs encoding FLAG-MS2-x4E-T fusion proteins (50 nl at ~500 ng/μl) were injected into stage VI oocytes, as described previously [27,28], with 150 oocytes injected for each mRNA. Oocytes were incubated for 36 h at 18^o^C before harvesting. Extracts were made by addition of 1 ml NET buffer and clarification by centrifugation at 10,000 g, 2 x 10 min, 4 ^o^C. To each extract were added 20 μl of Protein-G-Sepharose beads and 5 μl mouse monoclonal α-FLAG (Sigma; 4.2 mg/ml) antibody. The mixture was incubated for 4 h at 4 ^o^C, with constant rotation, and then centrifuged at 800 g, 2 min, 4 ^o^C, and the supernatant discarded. The beads were washed 3 x 1 ml NET buffer, and bound proteins eluted in 40 µl of PBS containing 250 µg/ml FLAG peptide. The eluate was concentrated in a micro-spin column (Pierce) and centrifuged at 13,000 rpm for 1min, before denaturation in SDS-Sample buffer and western blotting.

### Protein gel electrophoresis and western blotting

SDS-PAGE gels and western blotting were performed as described previously [28, 35]. Typically, 40 μg protein per lane was loaded. Primary antibodies and dilutions used were as follows: chicken eIF4E2 (1:2000; Novus Biologicals), rabbit eIF4E1 (1:15000; gift from Simon Morley), goat 4E-T (1:500; Abcam), mouse GFP (1:2000; Santa Cruz), rabbit p54/rck (Bethyl Labs; 1:2000), mouse CPEB [36] and rabbit MS2 antibodies [62]. Western blots were subsequently detected by enhanced chemiluminescence (ECL). Magic Mark XP Standard Western proteins (Invitrogen) were used as molecular weight markers.

## Results and Discussion

### Expression and quantitation of eIF4E1, eIF4E2 and 4E-T in HeLa and HEK293 cells

**Figure 1:**
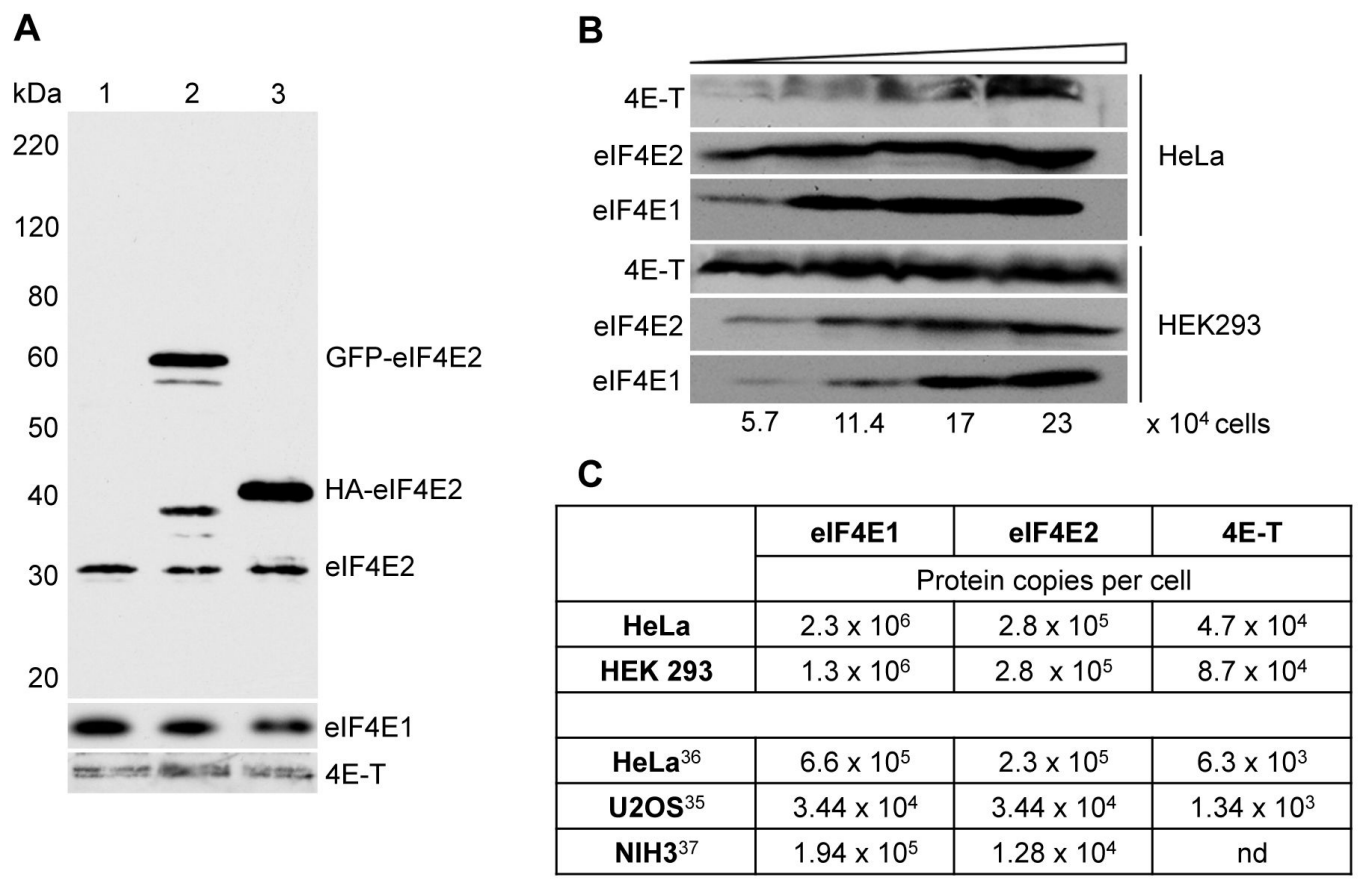
Quantitation of eIF4E1, eIF4E2 and 4E-T levels. **A**. Verification of chicken eIF4EL3/eIF4E2 antibody using untransfected Hela cells (1), and cells overexpressing GFP-eIF4E2 (2) and HA-eIF4E2 (3). Top panel developed with eIF4E2 antibody, bottom panels with eIF4E1 and 4E-T antibodies. Molecular weight standards are indicated in kDa. **B**. Comparison of eIF4E1, eIF4E2 and 4E-T levels in HeLa and HEK293 cells analysed by western blotting with indicated antibodies. Increasing amounts of lysate from HeLa cells (20, 40 and 80 µg total protein) and HEK293 cells (10, 20 and 40 µg) approximating to 5.7, 11.4, 17 and 23 x 10^4^ cells. **C**. Table comparing eIF4E1, eIF4E2 and 4E-T levels (protein copies/per cell) in HeLa and HEK293 determined by western blotting (top), to those reported in genome-wide mass spectrometry studies in HeLa [35], U20S [34] and NIH3T3 [36] cells.

To examine the expression of eIF4E2, we first identified an eIF4E2-specific antibody. Indeed, we found several commercial antibodies failing to detect this protein, with the exception of the chicken anti-eIF4EL3 antibody, which, as Figure 1A shows, detects both GFP-eIF4E2, HA-eIF4E2 and an endogenous protein of the predicted size of 28 kDa in HeLa cells. We compared the expression of eIF4E1, eIF4E2 and 4E-T in HeLa and HEK293 cells, probably the two most commonly used mammalian cell lines (Figure 1B and 1C). Levels of eIF4E1 and eIF4E2 are similar in both cell lines, with HeLa cells possessing slightly higher levels of eIF4E1 than HEK293 cells, though 4E-T was significantly more expressed in HEK293 than in HeLa cells. We also noted that in HEK293 cells 4E–T migrates solely as the lower band of the doublet observed in HeLa cells, possibly indicating differential phosphorylation in the two cell lines (Figure 1B).

Using a concentration series of recombinant human eIF4E1, eIF4E2 and 4E-T proteins made in *E. coli* alongside lysates prepared from HeLa and HEK293 cells in western blotting (data not shown), we estimated their levels in terms of protein copies per cell (summarised in Figure 1C). While these levels are higher than those obtained from recent proteome mass spectrometry determinations in U2OS, HeLa and NIH 3T3 cells and there are indeed considerable differences between these proteomic studies themselves [37–39], the following conclusions using all the data can be made. First, eIF4E1 levels are consistently higher than those of eIF4E2 (with the exception of U2OS cells where they are equal), and second, 4E-T levels are significantly much lower than either eIF4E protein. Our quantitation data show that eIF4E1:eIF4E2 ratios range from approx 8:1 to 5:1 in HeLa and HEK293 cells respectively, while ratios of eIF4E1:4E-T range from 50:1 to 15:1, and those of eIF4E2:4E-T vary from 6:1 to 3:1 (Figure 1C). We also considered the levels of eIF4G1+3 and 4E-BP1+2 in the proteomic studies [37–39], and concluded that 4E-T is the least expressed of these eIF4E-binding proteins in mammalian cells. Interestingly, too, none of these studies reported significant levels of eIF4E3. Altogether, these data indicate that eIF4E1 is the principal eIF4E protein in mammalian cells, and that eIF4E:4E-T ratios can vary from 3:1 to 50:1 depending on the cell and the cap-binding protein.

### eIF4E2 binds 4E-T via YX_4_LL and a nearby downstream sequence

**Figure 2:**
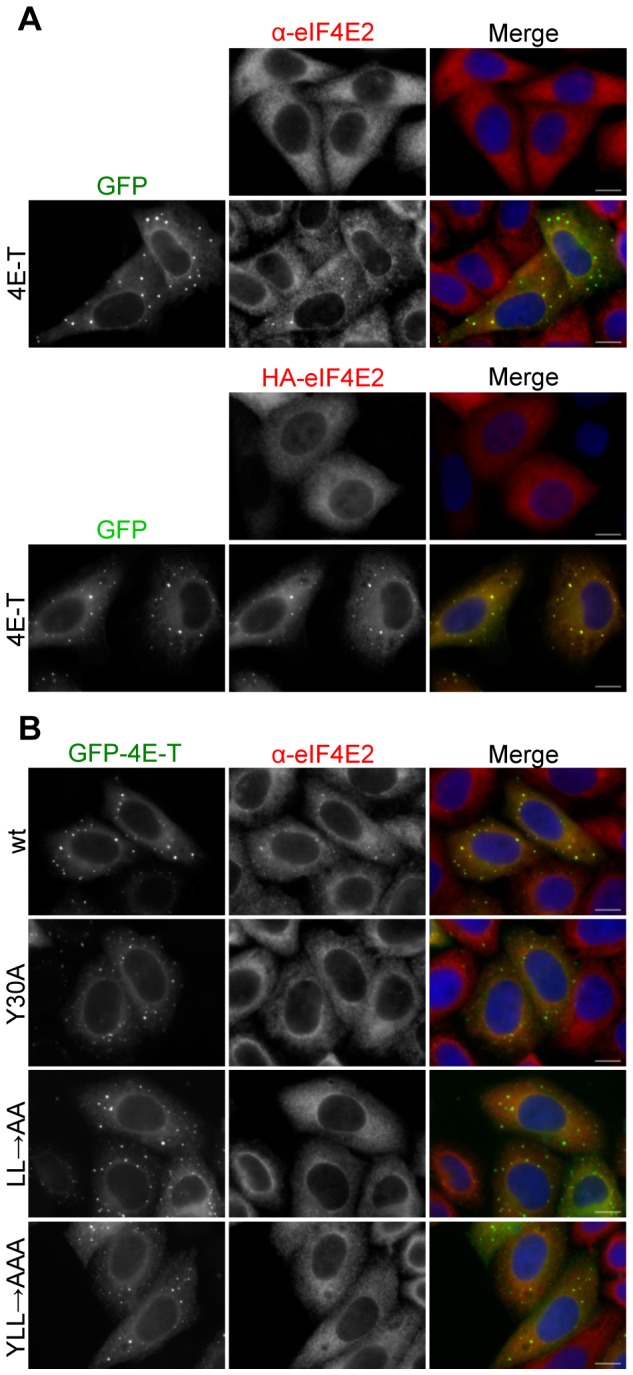
eIF4E2 is recruited to P-bodies by ectopic 4E-T. **A**. Cellular eIF4E2 distribution in HeLa cells was assessed by indirect immunofluorescence in the absence and presence of GFP-4E-T, and similarly for HA-tagged eIF4E2 co-transfected or not with GFP-4E-T. **B**. Wild-type but not mutant GFP-4E-T recruits eIF4E2 to P-bodies. Mutations in the 4E-T eIF4E-binding site residues Y^^30^^X_4_LL to alanine are indicated. Cells were also stained with DAPI. Scale bar, 10 µm.

eIF4E1 binding to 4E-T via its N-terminal YX _4_LL sequence has been reported previously, and shown to prevent interaction with eIF4G, reducing translation initiation of capped mRNAs [20,21,23,27]. Large scale yeast protein–protein interaction screens suggested 4E-T also interacts with eIF4E2 [40]. First, we verified that eIF4E2 binds 4E-T in the yeast two hybrid system. AH109 cells were transformed with pGADT7-4E-T and pGBKT7-4E2 plasmids and grown in -Leu/-Trp dropout medium to select for both plasmids. Stocks made from single colonies of double transformants were re-streaked on both -Leu/-Trp and -Ade/-His/-Leu/-Trp dropout plates to screen for plasmid retention and high-stringency protein–protein interactions, respectively. Yeast growth in stringent conditions was supported by 4E-T co-expressed with eIF4E2, with no growth in the case of controls (Figure S1A). The interaction was next assessed in HeLa cells over-expressing GFP-4E-T. In contrast to untransfected cells in which cellular eIF4E2 is essentially homogeneously distributed in the cytoplasm, ectopic 4E-T recruits eIF4E2 to P-bodies (Figure 2A), as was reported for tagged and cellular eIF4E1 ( [20,23], and Figure S2). We found that eIF4E2 co-localised with GFP-4E-T in P-bodies in approx. 70% of cells, relative to untransfected cells where it only co-localised with 4E-T P-bodies in 20% of cells, as verified by immunostaining. We confirmed this redistribution from the cytoplasm to P-bodies with HA-tagged eIF4E2, where it co-localises with GFP-4E-T in approx. 75% of transfected cells (Figure 2A). Moreover, we show that eIF4E2 does not localise to P-bodies containing GFP-4E-T with the mutations in the critical tyrosine and leucine residues of the Y^30^X_4_LL motif (Figure 2B and Figure S2), indicating that eIF4E2 associates with 4E-T in a similar manner as eIF4E1.

**Figure 3:**
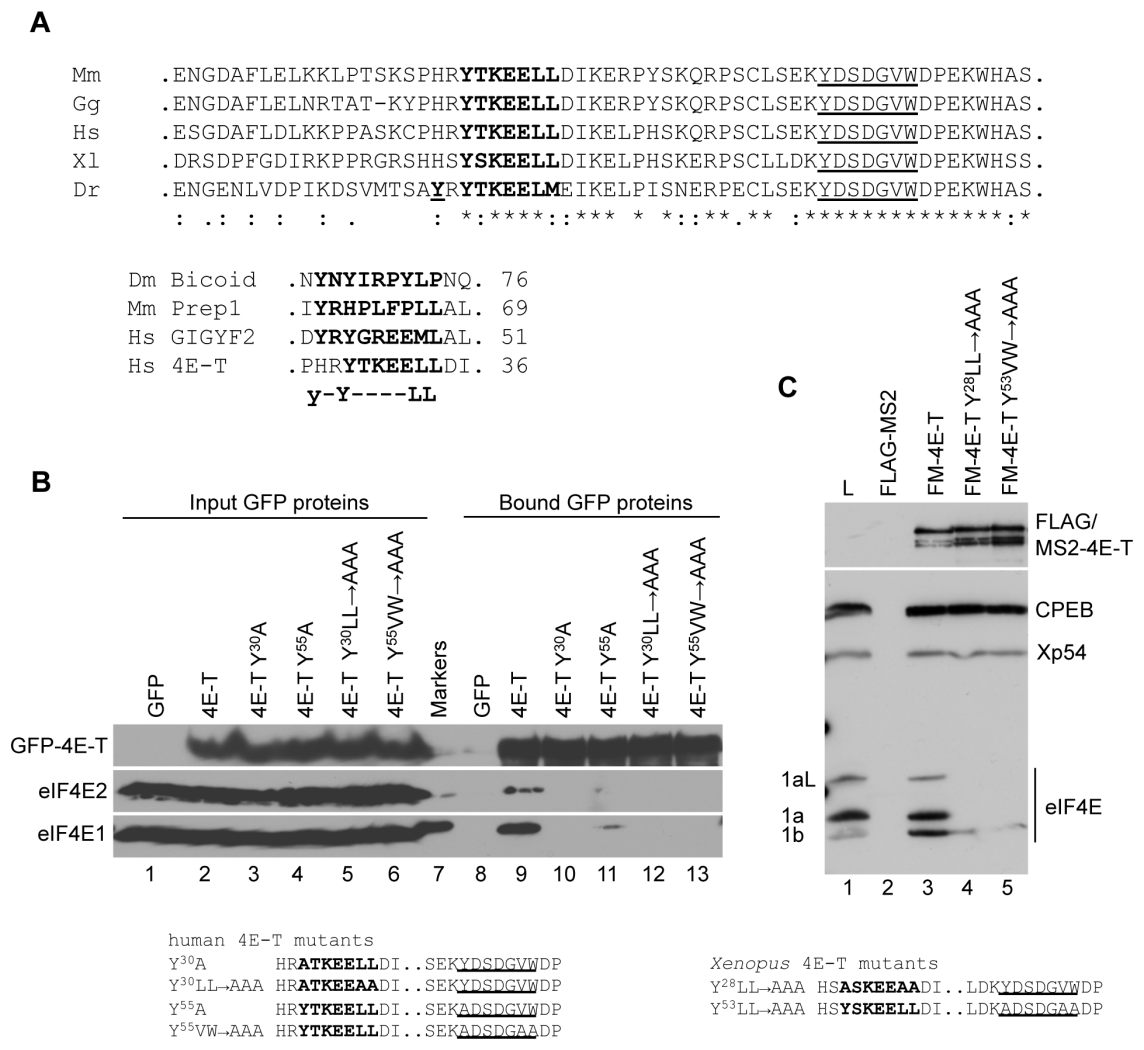
The eIF4E-binding site in 4E-T. **A**. Top. The sequences of the eIF4E2-binding site in Dm Bicoid [17], Mm Prep1 [18], Hs GIGYF2 [19] and Hs 4E-T ( [20]. Bottom. Alignments of five indicated vertebrate 4E-T sequences using ClustalW2. In bold are indicated the eIF4E-binding site residues, note the upstream tyrosine in Dr 4ET, bold and underlined. Underlined are the residues of a potential conserved second eIF4E-binding site. **B**. HEK293 cells were transfected with wild type or mutant GFP-4E-T as indicated and lysates were analysed by GFP-Trap and western blotting with eIF4E1, eIF4E2 and 4E-T antibodies. **C**. mRNAs encoding FLAG-MS2 and FLAG-MS2-X4E-T proteins were injected into *Xenopus* oocytes. After 16 hours, lysates were prepared and immunoprecipitated with FLAG antibodies, and the FLAG peptide eluates were analysed by western blotting with indicated antibodies. L lane indicates uninjected oocyte lysate. The mutations in human and *Xenopus* 4E-T sequences are indicated in B and C.

Previously characterised protein partners of eIF4E2/4EHP in fly and mammalian cells, including Bicoid [17], Prep1 [18] and GIGYF2 [41], were shown to interact via a slightly extended N-terminal binding sequence of YX _6_Lϕ or YXYX _4_Lϕ (Figure 3A). We thus examined whether 4E-T proteins possess this extended sequence. Interestingly, with the exception of several (but not all) fish 4E-T sequences (*Danio rerio*, *Oreochromis* *niloticus* and *Oryzias latipes*, though not *Takifugu* *rubripes* ), which contained an additional tyrosine at -2 position relative to the consensus tyrosine, all other vertebrates lacked this upstream residue. Examining the sequence of 4E-T, we noted a possible nearby downstream eIF4E-binding site Y^55^X_4_VW, that is conserved in vertebrates, including *Xenopus* (Figure 3A). While this putative additional sequence is not strictly speaking a consensus sequence, we were intrigued by the potential of sequences downstream of the consensus site in enhancing eIF4E-binding, noted in both 4E-BP and eIF4G [42,43].

To assess this possibility, we employed a pull-down assay with lysates from HEK293 cells overexpressing GFP-4E-T using the GFP-Trap [44], as a more sensitive method to detect interactions in mammalian cells than the P-body recruitment approach. First, we confirmed that eIF4E2 binds GFP-4E-T, and comparison of input and immunoprecipitated levels indicates that eIF4E2 binds with apparent lower affinity to 4E-T than does eIF4E1 (Figure 3B, lanes 2 and 9). Furthermore, in a reciprocal immunoprecipitation, HA-eIF4E2 brings down endogenous 4E-T (Figure S1B). Therefore in mammalian cells, 4E-T interacts with eIF4E2 as well as eIF4E1. Moreover, mutation of the critical conserved tyrosine and leucines in Y^30^X_4_LL or just the tyrosine on its own to alanine abrogated the interaction between GFP-4E-T and eIF4E2, in agreement with the HeLa P-body data (Figure 2B), as well as between eIF4E1 and GFP-4E-T (Figure 3B, lanes 10 and 12). We next tested whether the Y^55^X_4_VW binding site contributes to eIF4E binding. Mutation of Y^55^ to alanine reduced binding of eIF4E1 to 4E-T, and all interaction was lost with the triple mutation of Y^55^VW^^60/61^^ positions, while no eIF4E2 binding was detected with either mutant 4E–T protein (Figure 3B, lanes 11 and 13). In agreement with these pull-down results, the single tyrosine mutation (Y^55^ to A) in GFP-4E-T expressed in HeLa cells largely unaffected eIF4E1 localisation in P-bodies (wt and Y^55^ both approx. 90% of cells with P-bodies) while preventing that of eIF4E2 (wt and Y^55^ approx. 70 and 10 % of cells respectively with P-bodies; Figure S2).

Altogether, our data thus suggests that while both eIF4E1 and eIF4E2 interact with sequences downstream of Y^30^X_4_LL of 4E-T, the contribution of the downstream sequence is less important in the case of eIF4E1, indicating that despite the participation of the same region of 4E-T, formation of 4E-T:eIF4E1/2 complexes is differentially stabilised.

### In Xenopus oocytes, eIF4E1 binding to 4E-T is also sensitive to the same downstream mutations

We next examined the interaction between 4E-T and eIF4E proteins in *Xenopus* oocytes. FLAG-MS2-tagged x4E-T and the control FLAG-MS2 protein were expressed from *in vitro* transcribed mRNA microinjected into stage VI oocytes, and co-precipitation of endogenous eIF4E1 proteins by FLAG antibodies was assessed by western blotting. Unlike endogenous 4E-T, ectopic 4E-T can bind both short and long forms of eIF4E1 (differing in an 18 amino acid repeat) [27], though it preferentially interacts with eIF4E1b, as shown by comparing the input and the wild-type protein bound lanes (Figure 3C, lanes 1 and 3). Mutation of the consensus eIF4E-binding site, Y^28^LL^^33/34^^ to AAA in FLAG-MS2-4E-T abolishes all eIF4E1a binding as does mutation of the second putative site Y^53^VW^^58/59^^ to AAA, while both sets of mutations also abolish most, though not all, eIF4E1b binding (Figure 3C, lanes 4 and 5). Neither set of mutations abrogates 4E-T co-immunoprecipitating CPEB or Xp54 helicase, demonstrating that they do not result in extensive misfolding. We could not assess *Xenopus* 4E-T binding to eIF4E2, since the chicken eIF4EL3 antibody does not detect an oocyte protein of the appropriate size, though we suspect eIF4E2 to be maternally expressed, as it is in mice [18].

Altogether we conclude that sequences downstream of the canonical eIF4E-binding site in human or *Xenopus* 4E-T, including the YX _4_VW motif, strongly influence 4E–T interaction with eIF4E1 or eIF4E2 isoforms and may regulate their differential binding. The exact delineation of the additional sequences is beyond the scope of this study, and clearly warrants further investigation, ideally at the structural level. We note also that the previously described downstream eIF4E-interacting sequences in human 4E-BP and eIF4G proteins [42,43], located approx. 25 away from the consensus sequence, are not conserved in sequence, and are not present in 4E-T. Moreover, the downstream sequences may not necessarily interact directly with eIF4E, but could enhance the ability of the canonical consensus site to form a stable α-helix [45]. Indeed, secondary structure predictions for human or *Xenopus* 4E-T (using Jpred3 server: http://www.compbio.dundee.ac.uk/www-jpred/) suggests that only the canonical binding site Y(T/S) KEELL has the potential to form an α-helix, not YDSDGVW, though this does not exclude it from directly binding eIF4E. Interestingly, the *D. melanogaster* protein Cup, related to human 4E–T, has two nearby eIF4E-binding sites, the high-affinity one which conforms to the consensus, being Y^342^TRSRLM, with a second lower affinity and non-consensus one, ELEGRLR, some 30 residues downstream [34]. The second site binds laterally and perpendicularly in α-helix form to the eIF4E β-sheet [46], and plays a role in the stabilisation of associated mRNA [47].

### Unlike eIF4E1, eIF4E2 does not re-localise to stress granules in arsenite-treated cells

**Figure 4:**
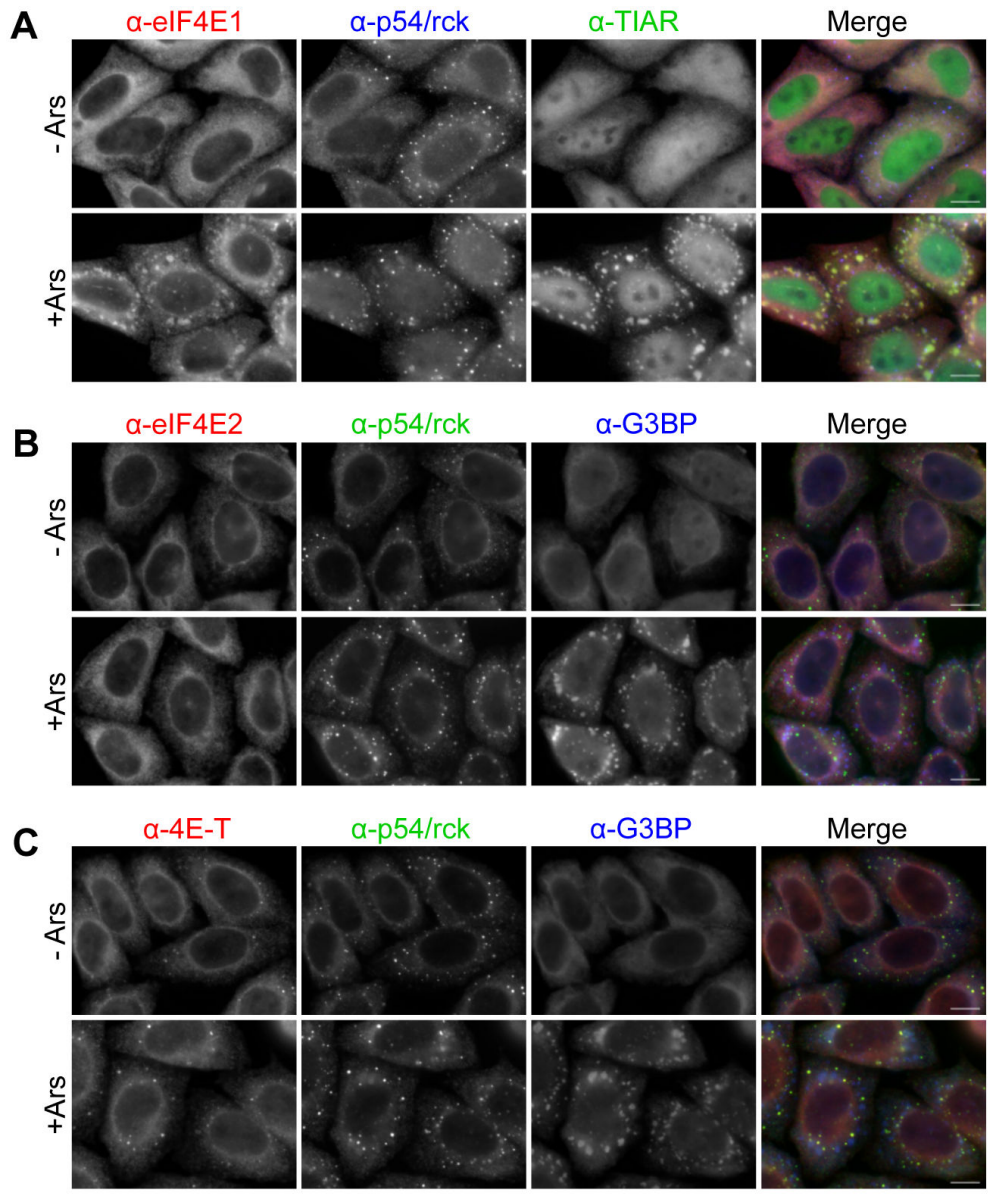
eIF4E1 and eIF4E2 distribution in arsenite-treated cells. Control HeLa cells (- Ars) and cells treated with arsenite (+ Ars) to induce stress granules were immunostained with eIF4E1 (**A**), eIF4E2 (**B**) and 4E-T (**C**) antibodies, and counter-stained with antibodies against p54/rck (P-body marker), and TIAR or G3BP (stress granule markers) as indicated. Scale bar, 10 µm.

In addition to its native localisation in P-bodies, eIF4E1 is found in stress granules in mammalian cells treated briefly with arsenite, resulting in oxidative stress [48]. Stress granules are large cytoplasmic granules, considerably larger than P-bodies, which contain stalled translation initiation complexes including eIF4E, eIF4A, eIF4G and PABP, non-translating mRNA and RNA-binding proteins such as G3BP and TIAR [36,48,49]. Like P-bodies, stress granules are dynamic complexes whose assembly is dependent on the pool of nontranslating mRNAs, and indeed the two types of mRNP granules are frequently found juxtaposed or overlapping [49,50]. Arsenite-treated HeLa cells contain typical TIAR and G3BP-containing stress granules, to which eIF4E1 co-localises (Figure 4A), as shown by immunostaining. Interestingly, endogenous p54/rck remains in P-bodies adjacent to stress granules in these cells, as observed previously, though RFP-p54/rck can relocate to stress granules from the the diffuse cytoplasmic pool [48,50]. In contrast to eIF4E1, eIF4E2 remains homogenously distributed in the cytoplasm in arsenite-treated cells (Figure 4B), and 4E-T remains partly cytoplasmic and partly enriched in P-bodies, as in untreated cells (Figure 4C). In other words, localisation of eIF4E2 and 4E-T is insensitive to arsenite, unlike eIF4E1, implying distinct functions to translation initiation.

### Inhibition of RNA transcription does not impact on eIF4E2 distribution, but enhances eIF4E1 localisation to P-bodies

**Figure 5:**
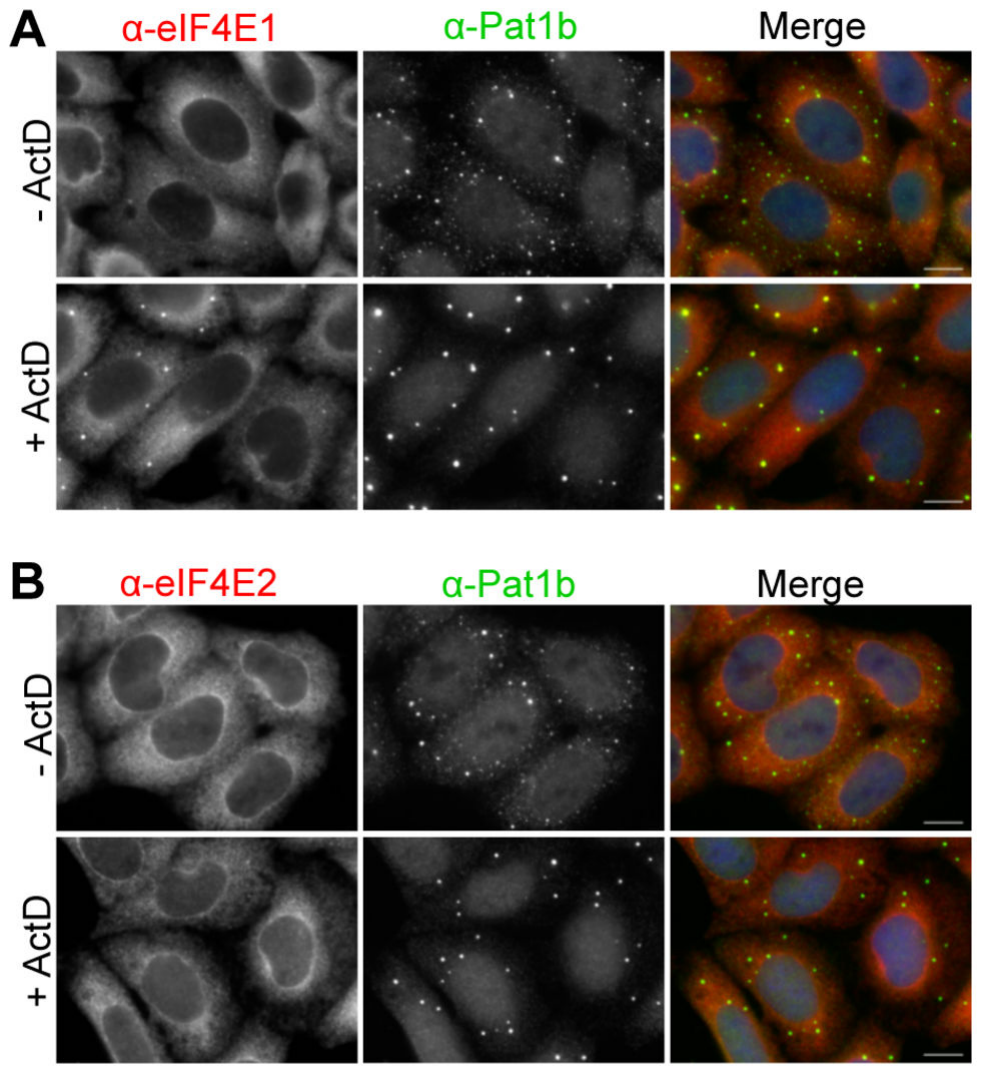
eIF4E1 and eIF4E2 distribution in Actinomycin D-treated cells. Control HeLa cells (- ActD) and cells treated with Actinomycin D (+ ActD) to inhibit RNA synthesis were immunostained with eIF4E1 (**A**) and eIF4E2 (**B**) antibodies, and counter-stained with Pat1b antibodies. Note that the apparent nuclear staining in the case of Pat1b antibody was previously shown to be insensitive to Pat1b siRNA [49]. Cells were also stained with DAPI. Scale bar, 10 µm.

We recently showed that in HeLa cells treated with Actinomycin D for 5 hours to inhibit new RNA synthesis, the P-body factors Pat1b, p54/rck and 4E-T were localised in fewer but bigger P-bodies than in untreated HeLa cells [51]. Particularly striking was the loss of small P-body foci upon treatment, suggesting that in the absence of new transcripts, smaller foci aggregate into larger ones. Moreover, P-bodies in *Trypanosoma brucei* also enlarge upon Actinomycin D treatment [52]. The cause of this increase is not clear but may be due to the reduced levels of newly synthesised mRNAs resulting in an inhibition of protein synthesis and thus an increase in P-bodies. Similarly, using Pat1b immunostaining as a control, we note that eIF4E1 redistributes to P-bodies in ActD-treated HeLa cells, even in the absence of ectopic 4E-T (Figure 5A). In contrast, eIF4E2 localisation in the cell is unaffected by this treatment (Figure 5B), additionally highlighting the differences between eIF4E1 and eIF4E2.

### eIF4E2 is a 4E-T-independent nucleocytoplasmic shuttling protein

**Figure 6:**
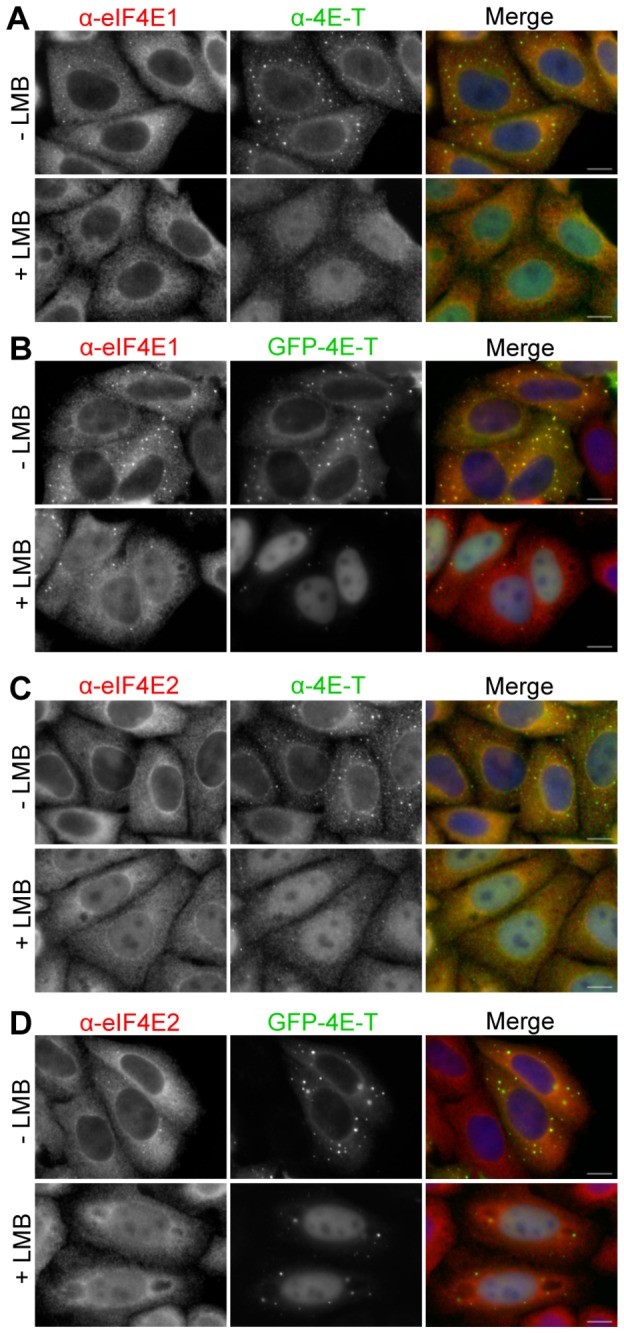
eIF4E2 is a nucleocytoplasmic shuttling protein. Hela cells were treated with Leptomycin B for 5 hrs (+ LMB) or with methanol as control (- LMB), without (**A** and **C**) and with ectopic GFP-4E-T (**B** and **D**), and immunostained with eIF4E1 (**A** and **B**), eIF4E2 (**C** and **D**) and 4E-T antibodies, while GFP-4E-T fluorescence was detected directly in transfected cells. Scale bar, 10 µm.

Previously, 4E-T was shown to transport eIF4E1 into nuclei in cells treated with Leptomycin B (LMB), which inhibits the Crm1 receptor [53], demonstrated with tagged proteins [20]. Indeed, these studies defined 4E-T as an eIF(4E-T) ransport protein. Here we demonstrate that while 4E-T (both cellular and ectopic) is nuclear in treated cells attesting to the LMB addition, eIF4E1 is unresponsive to LMB in untransfected HeLa cells, and only becomes sequestered within nuclei in the presence of both GFP-4E-T and LMB (Figure 6A and B). 4E-T is a nucleocytoplasmic shuttling protein, which does not require eIF4E-binding to translocate to nuclei in LMB-treated cells ( [20,51], Figure S3), whereas eIF4E1 requires additional 4E-T protein to visibly accumulate in nuclei in these conditions (Figure 6). This observation presumably reflects the relatively low levels of 4E-T to eIF4E1 in HeLa cells (Figure 1).

**Figure 7:**
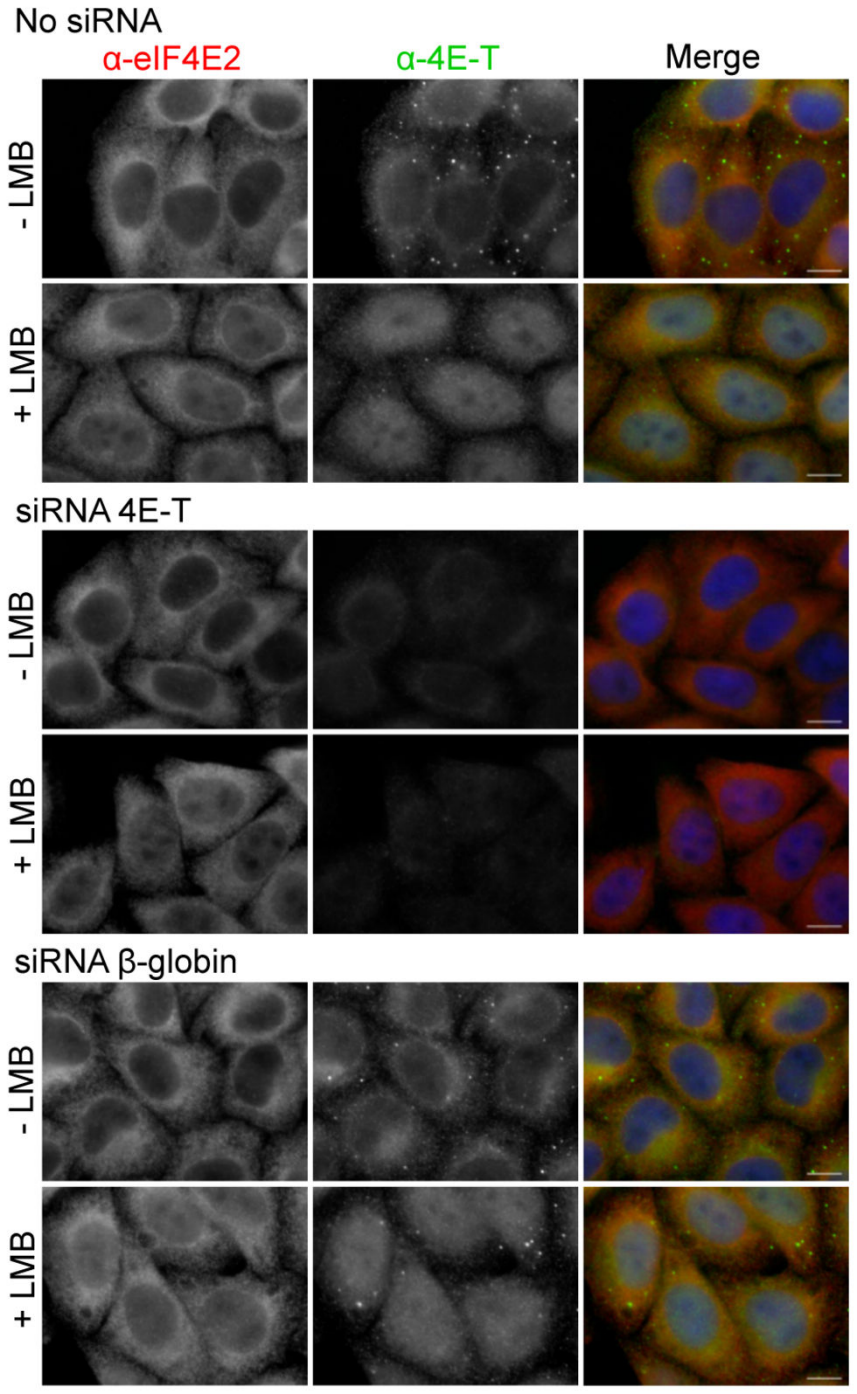
eIF4E2 shuttling in the presence of LMB is 4E-T independent. Untransfected Hela cells (no siRNA), or cells transfected with 4E-T siRNA or control β-globin siRNA were treated with LMB and immunostained with eIF4E2 and 4E-T antibodies as indicated. Cells were also stained with DAPI. Scale bar, 10 µm.

In contrast, eIF4E2 becomes partially nuclear in LMB-treated cells, in the absence of ectopic 4E-T, and overexpression of 4E-T does not enhance this nuclear accumulation (Figure 6C and D). To confirm this finding, 4E-T was depleted from cells using RNAi, which were subsequently treated with LMB and immunostained for eIF4E1, eIF4E2 and 4E-T. 4E-T siRNA was shown to be effective in depleting cellular 4E-T, unlike the control β-globin siRNA (lacking a target in HeLa cells). Nevertheless, eIF4E2 continues to accumulate in nuclei in LMB-treated 4E-T depleted cells, and as predicted from above, eIF4E1 remains cytoplasmic (Figure 7 and Figure S4. Thus, 4E-T is a transporter for eIF4E1 only, and not for eIF4E2, though 4E-T binds both proteins, and ectopic 4E-T relocalises eIF4E2 to P-bodies (Figures 2 and 3; Figures S1 and S2). Previously we reported that in approx. 33% of transfected and LMB-treated cells GFP-4E-T localises in numerous foci, overlapping or close to PML bodies [49]. Here we show that the Y^30^A mutation, which in such cells would only prevent eIF4E1 import and not that of eIF4E2, strikingly enhances the number and brightness of such GFP-4E-T foci, though the reasons for this are not known (Figure S3).

**Figure 8:**
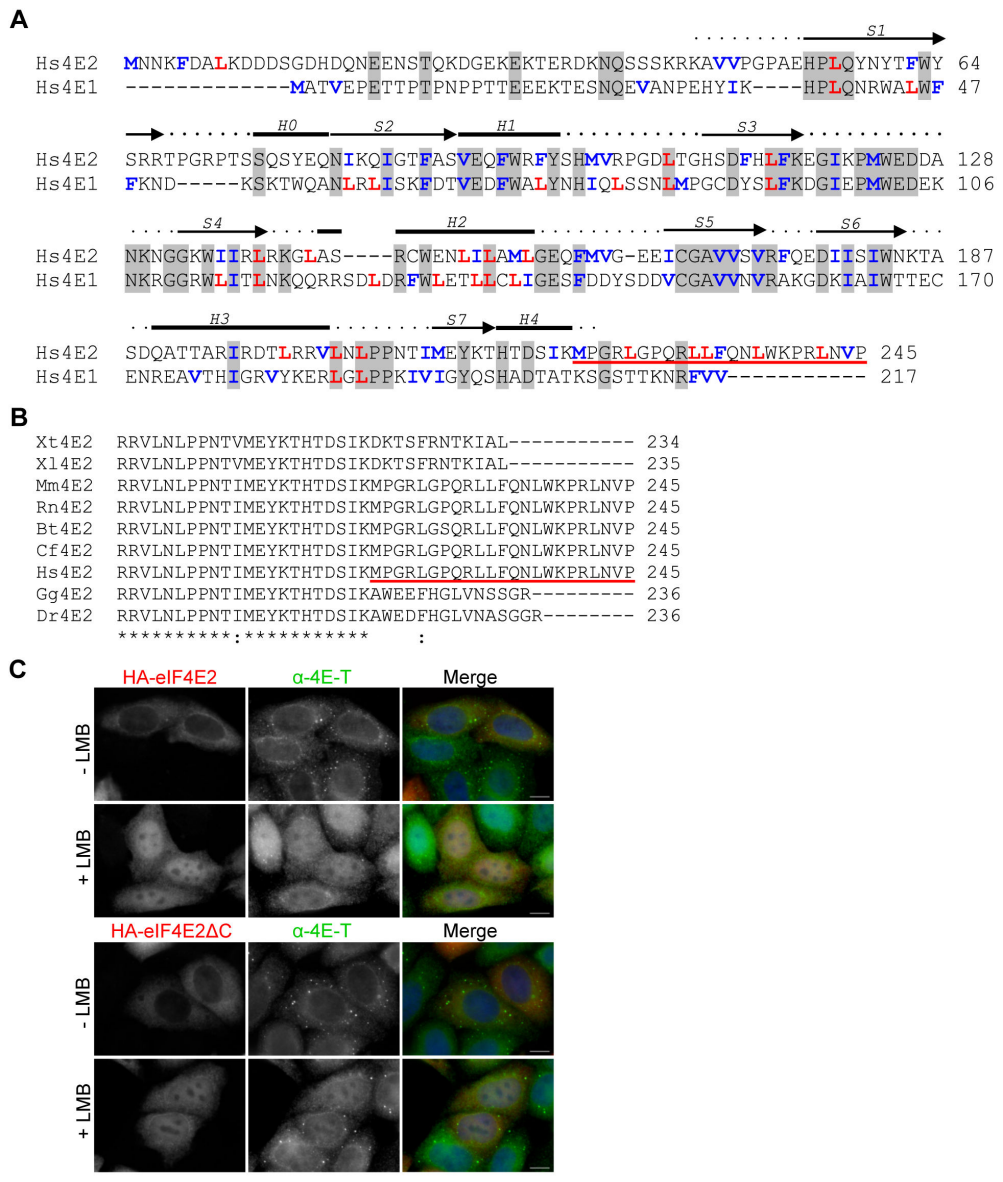
The leucine-rich C-terminus of eIF4E2 does not act as a NES. **A**. Sequence alignment of human eIF4E1 and eIF4E2 performed with ClustalW2. Secondary structural elements of α-helices (H0-H4) and β-strands (S1-S7) were shown above the alignment according to the crystal structure of human eIF4E2 in complex with m^7^ GTP (PDB id: 2JGB) [15]. Identities are shaded in grey. With blue and red are marked hydrophobic residues that typically form the NES. The leucine-rich C-terminus of eIF4E2 is underlined in red. **B**. ClustalW alignment of the C-terminal regions of indicated eIF4E2 proteins. **C**. HeLa cells transfected with HA-eIF4E2 full-length protein or HA-eIF4E2ΔC (truncated at residue 222) were treated with LMB or methanol (-) for 5 hrs and immunostained with HA and 4E-T antibodies as indicated. Cells were also stained with DAPI. Scale bar, 10 µm.

How eIF4E2 enters and exits nuclei remains to be investigated, whether on its own or with a shuttling partner protein. We noted that mammalian eIF4E2 proteins possess short N- and C-terminal extensions relative to eIF4E1 proteins, and that its C-terminal region (in human protein) contains five conserved leucines, possibly related to the nuclear export signals (NES) in Crm1-dependent shuttling proteins. Most NES sequences conform to the consensus ϕ-(x)_2-3_-ϕ-(x)_2-3_-ϕ-x-ϕ, where x is an amino acid that is preferentially charged, small or polar, and ϕ is hydrophobic [54]. However the spacing of the leucines in the eIF4E2 C-terminal portion does not follow the consensus, and they are not conserved in vertebrates (Figure 8A and B). More importantly, truncation of HA-tagged eIF4E2 at amino acid 222 did not lead to eIF4E2 accumulation in nuclei in the absence of LMB (Figure 8C), demonstrating that they do not act as an NES. eIF4E1 localisation can also be dictated by 4E-BPs [55], and this may be the case for eIF4E2 as well. Irrespective of the shuttling mechanism, it is interesting to note yet another difference between eIF4E1 and eIF4E2, and to speculate on their nuclear functions, perhaps in specific mRNA export [56].

## Conclusions and Implications

We examined the expression levels, 4E-T binding and localisation of eIF4E2, a close homologue of eIF4E1, but which binds the cap and eIF4G weakly, and which appears to function rather as a regulator of translation. We observe that levels of eIF4E1 exceed those of eIF4E2 some 5-10 x in mammalian cells, and that both proteins interact with the low abundance 4E–T protein via its N-terminal YX _4_Lϕ consensus eIF4E-binding sequence also present in eIF4G and 4E-BPs. Our evidence for 4E-T:eIF4E2 binding was obtained in the Y2H system, in HeLa cells with ectopic 4E-T recruiting eIF4E2 to P-bodies, and in pull-down assays with GFP-4E-T. Examining the sequences surrounding YX _4_Lϕ suggested that downstream sequences, possibly involving a look-alike second motif YX _4_VW, were also involved in eIF4E1 and eIF4E2 binding, adding to our understanding of extended sequences that influence eIF4E interactions with eIF4G and 4E-BP proteins.

To our knowledge this is the first study reporting cellular eIF4E2 distribution. In contrast to eIF4E1, eIF4E2 was unresponsive to both arsenite and Actinomycin D treatment in terms of its localisation, and was observed to shuttle with LMB in the absence of 4E-T. Altogether we conclude that these two proteins, while clearly related at the sequence level, are likely to participate in distinct cellular processes. It is intriguing to note that RNAi-mediated depletion of eIF4E2 in U87MG glioblastoma cells in normal conditions [57] has no significant effect on total protein synthesis, while in HeLa cells, an activation of approx. 30% in translation rates was reported [19], indicating a general negative role in translation. On the other hand, depletion of eIF4E1, counterintuitively not apparently affecting global protein synthesis very much, leads to the degradation of hypophosphorylated 4E-BP, thus allowing the remaining low eIF4E protein levels following RNAi-silencing to continue supporting translation initiation [58]. Since both eIF4E proteins have been captured in global screens of RNA-binding proteins in UV cross-linked mammalian cells, as has 4E-T [59,60], it will be of interest in future investigations to assess the relative binding of eIF4E proteins and 4E-T to mRNA. It will also be of interest to examine to what extent their relative levels and/or activities change in different conditions, during the cell cycle or in response to signalling, for example. It is already clear that eIF4E and 4E-T are subject to phosphorylation [1,41], which may affect their interactions with mRNA or RNA-binding proteins, and eIF4E2 can be modified by ISG15 ubiquitylation shown to significantly enhance its cap structure-binding [61,62]. And lastly, since eIF4G dramatically enhances eIF4E interaction with the mRNA 5′-cap [63], perhaps Bicoid, GIGYF2 or 4E-T may similarly regulate eIF4E2 cap-binding. Indeed, it is no doubt noteworthy that eIF4E2 (and eIF4E1b), negative regulators of translation and components of RNA-binding complexes (see Introduction), bind the cap less efficiently than eIF4E [16,27], allowing them on the one hand to interact with the cap by virtue of being tethered to particular mRNAs, and to be released from the cap on the other hand in response to cues disassembling the complex, without impeding general protein synthesis, and with the potential to be regulated by binding proteins. 

## Supporting Information

Figure S1 **eIF4E2 interacts with 4E-T.** **A**. Interaction shown in yeast two hybrid assay. Growth of indicated yeast bait and prey vectors in medium (SD/-Leu/-Trp) and high stringency (SD/-Ade/-His/-Leu/-Trp) plates. **B**. Interaction shown in pull-down assay in HeLa cell lysates overexpressing HA-eIF4E2, immunoprecipitated with eIF4E2 antibodies. Lanes: 0.5, 1, 2 μg α-eIF4E2 – resin incubated with given amount of α-eIF4E2, L – HeLa cells lysate, C – control resin without α-eIF4E2.
(TIF)

Figure S2 **Differential effects of Y^55^A mutation in GFP-4E-T on P-body recruitment of eIF4E1 and eIF4E2.** Cellular eIF4E1 and eIF4E2 distribution in HeLa cells was assessed by indirect immunofluorescence in the presence of wild-type GFP-4E-T, and its Y^30^A and Y^55^A mutant versions. Red boxes indicate eIF4E1 P-body staining in cells transfected with GFP-4E-T-Y^55^ A. Scale bar, 10 µm.
(TIF)

Figure S3 **The Y^30^A mutation in GFP-4E-T enhances its localisation to nuclear foci in LMB-treated cells.** Cellular distribution of eIF4E1 in HeLa cells transfected with wild-type GFP-4E-T, and its Y^30^A mutant version, and treated with LMB (+) or methanol vehicle (-). Cells were also stained with DAPI. Scale bar, 10 µm.
(TIF)

Figure S4 **4E-T depletion does not affect eIF4E1 localisation in the presence of LMB.** Untransfected Hela cells (no siRNA), or cells transfected with 4E-T siRNA or control β-globin siRNA were treated with LMB and immunostained with eIF4E1 and 4E-T antibodies. Cells were also stained with DAPI. Scale bar, 10 µm.
(TIF)

Table S1 **Oligonucleotide sequences used for cloning and mutagenesis.**
(DOC)
