# Correction: Investigating the Consequences of eIF4E2 (4EHP) Interaction with 4E-Transporter on Its Cellular Distribution in HeLa Cells

**DOI:** 10.1371/annotation/da84d2d8-fba4-409d-9063-2ef01385eecc

**Published:** 2013-11-06

**Authors:** Dorota Kubacka, Anastasiia Kamenska, Helen Broomhead, Nicola Minshall, Edward Darzynkiewicz, Nancy Standart

In the caption of Figure 1, reference [35] should be [38], [34] should be [37] and [36] should be [39].

In the caption of Figure 5, reference [49] should be [35].

In the section "elF4E2 binds 4E-T via YX_4_LL and nearby downstream sequence" of the Results and Discussion, reference [41] should be [19]. 

