# Supplementary figures and images for: Investigating the Consequences of eIF4E2 (4EHP) Interaction with 4E-Transporter on Its Cellular Distribution in HeLa Cells
Source: PLoS One. 2013 Aug 21;8(8):e72761. doi: 10.1371/journal.pone.0072761 (PMC3749138; doi:10.1371/journal.pone.0072761)

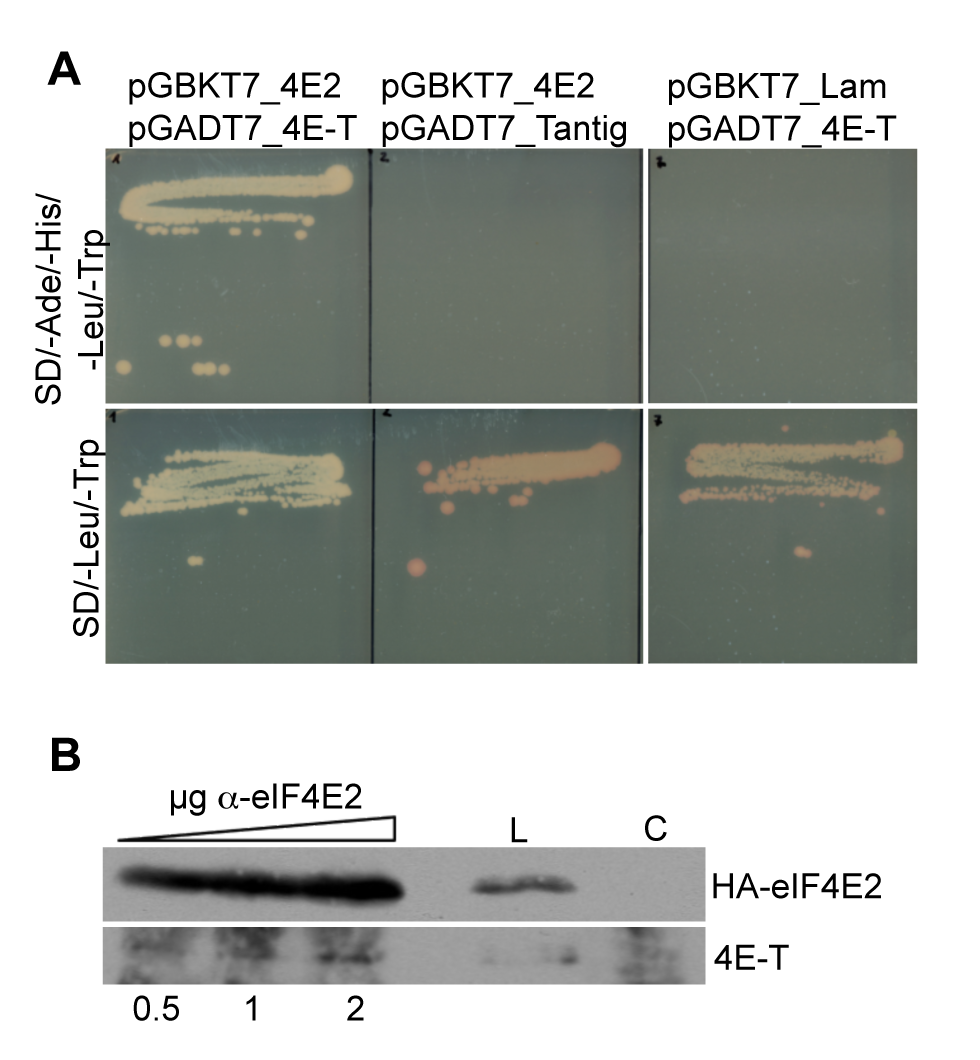

Supplement: Figure S1 — eIF4E2 interacts with 4E-T. A. Interaction shown in yeast two hybrid assay. Growth of indicated yeast bait and prey vectors in medium (SD/-Leu/-Trp) and high stringency (SD/-Ade/-His/-Leu/-Trp) plates. B. Interaction shown in pull-down assay in HeLa cell lysates overexpressing HA-eIF4E2, immunoprecipitated with eIF4E2 antibodies. Lanes: 0.5, 1, 2 μg α-eIF4E2 – resin incubated with given amount of α-eIF4E2, L – HeLa cells lysate, C – control resin without α-eIF4E2. (TIF) [file pone.0072761.s001.tif]

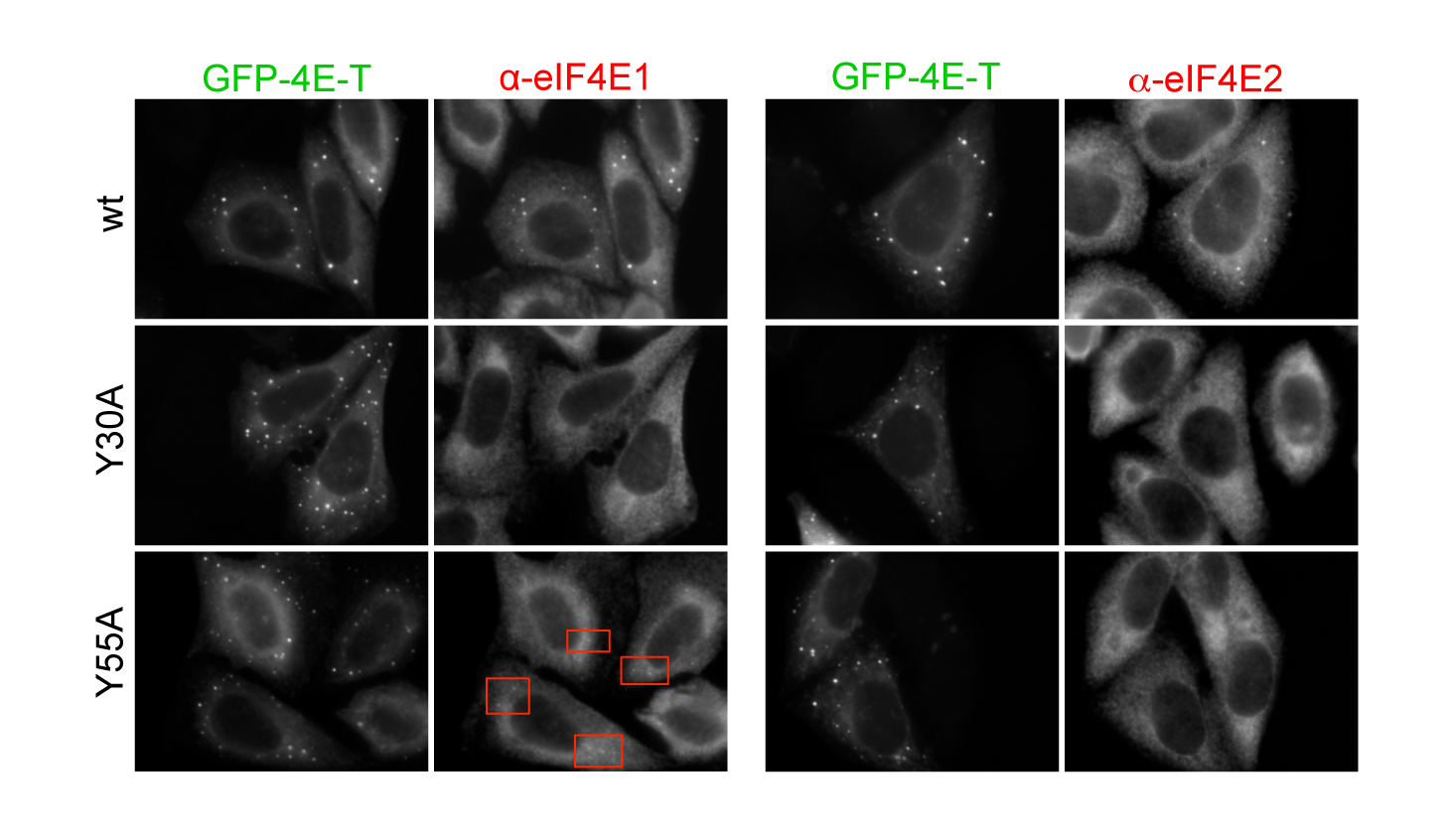

Supplement: Figure S2 — Differential effects of Y55A mutation in GFP-4E-T on P-body recruitment of eIF4E1 and eIF4E2. Cellular eIF4E1 and eIF4E2 distribution in HeLa cells was assessed by indirect immunofluorescence in the presence of wild-type GFP-4E-T, and its Y30A and Y55A mutant versions. Red boxes indicate eIF4E1 P-body staining in cells transfected with GFP-4E-T-Y55 A. Scale bar, 10 µm. (TIF) [file pone.0072761.s002.tif]

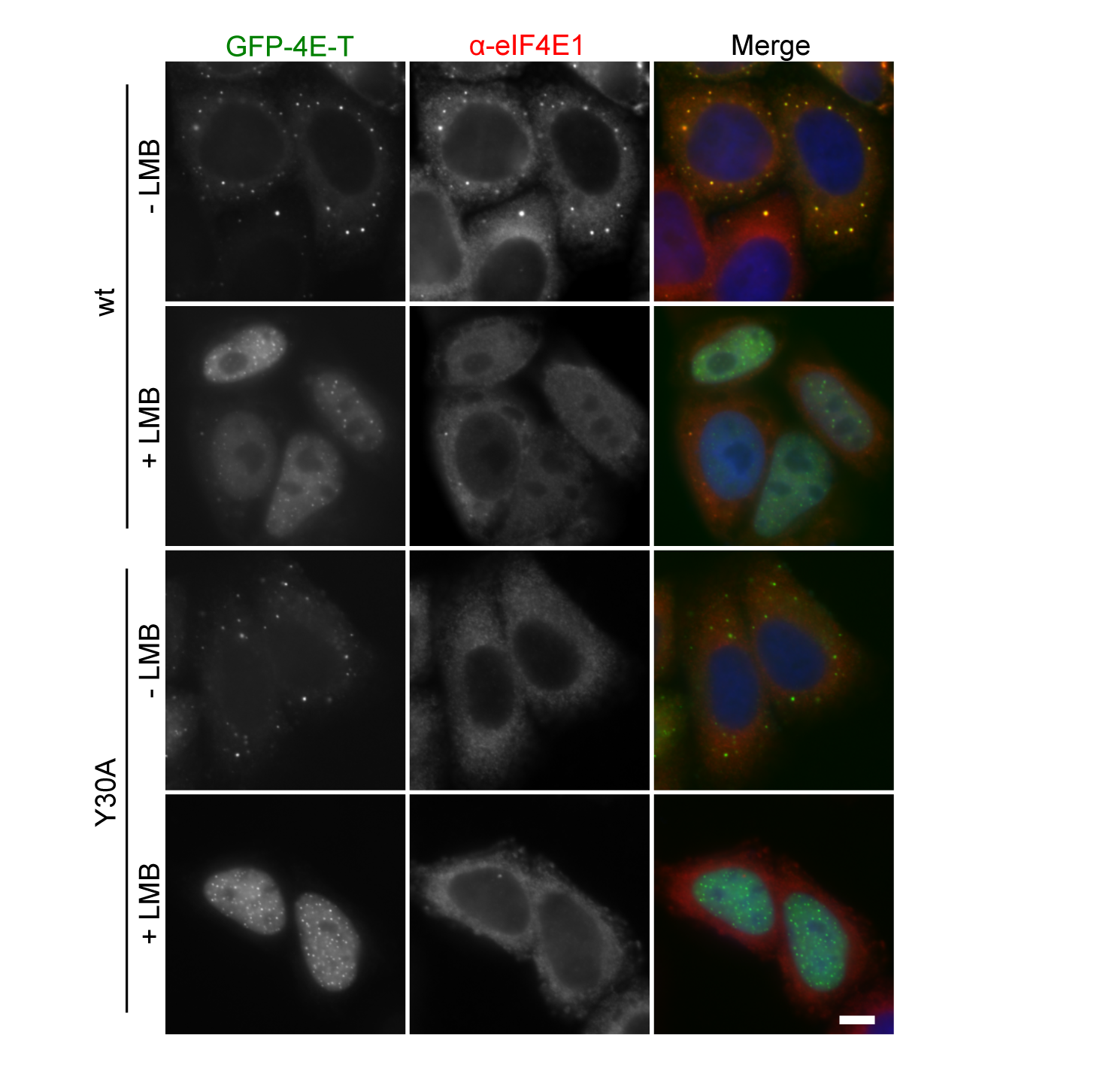

Supplement: Figure S3 — The Y30A mutation in GFP-4E-T enhances its localisation to nuclear foci in LMB-treated cells. Cellular distribution of eIF4E1 in HeLa cells transfected with wild-type GFP-4E-T, and its Y30A mutant version, and treated with LMB (+) or methanol vehicle (-). Cells were also stained with DAPI. Scale bar, 10 µm. (TIF) [file pone.0072761.s003.tif]

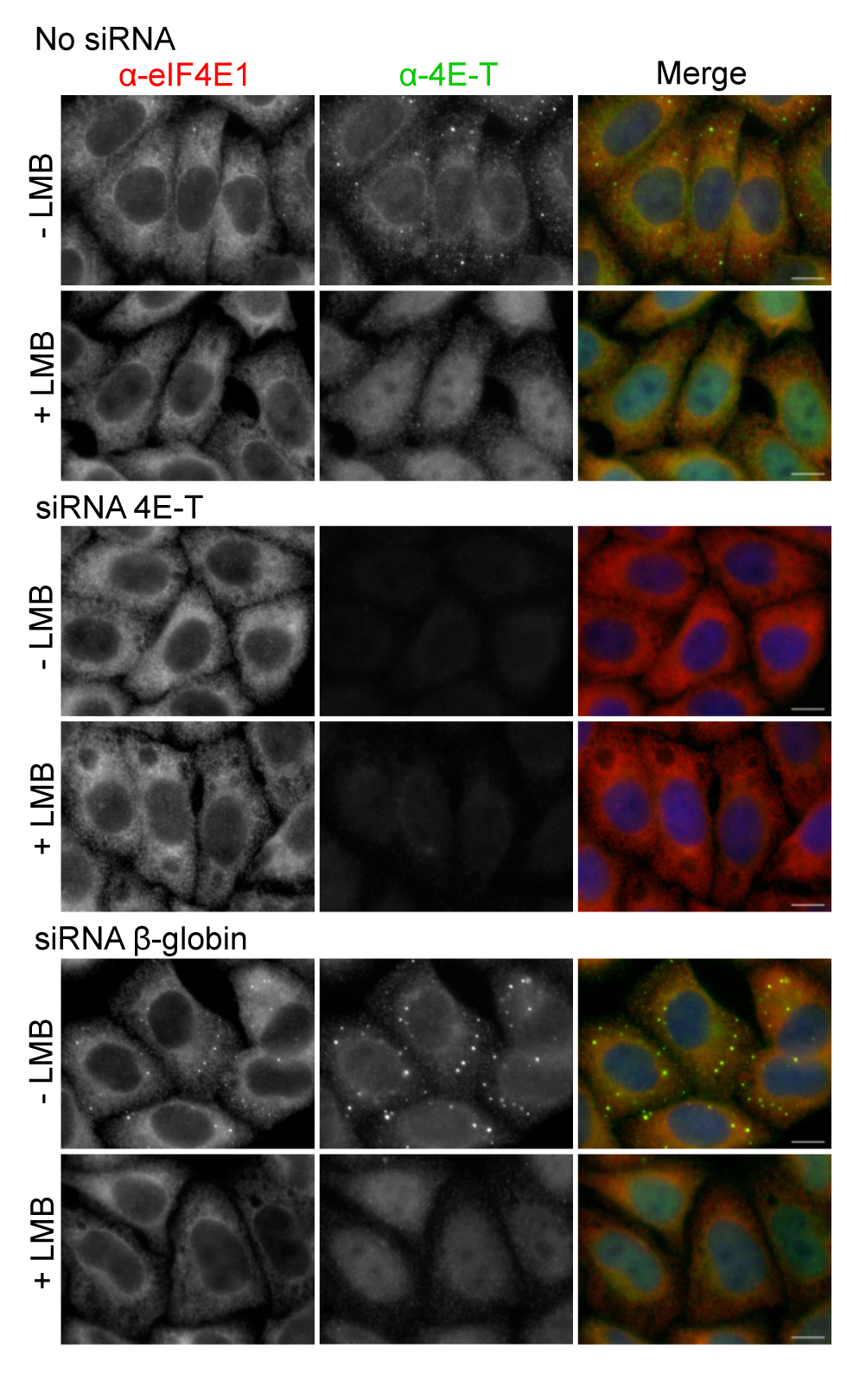

Supplement: Figure S4 — 4E-T depletion does not affect eIF4E1 localisation in the presence of LMB. Untransfected Hela cells (no siRNA), or cells transfected with 4E-T siRNA or control β-globin siRNA were treated with LMB and immunostained with eIF4E1 and 4E-T antibodies. Cells were also stained with DAPI. Scale bar, 10 µm. (TIF) [file pone.0072761.s004.tif]
