# Supplementary material for: Investigating the Consequences of eIF4E2 (4EHP) Interaction with 4E-Transporter on Its Cellular Distribution in HeLa Cells
Source: PLoS One. 2013 Aug 21;8(8):e72761. doi: 10.1371/journal.pone.0072761 (PMC3749138; doi:10.1371/journal.pone.0072761)
Supplement: Table S1 — Oligonucleotide sequences used for cloning and mutagenesis. (DOC) [file pone.0072761.s005.doc]

**Table 1.** Oligonucleotide sequences used for cloning and mutagenesis

| Primer name | Vector | Sequences 5’-3’ |
| --- | --- | --- |
| FLAGNcoIFwd  FLAGNcoIRev | pGEM T-easy+  3x FLAG (NcoI) | ctcgatccatggattataaggat  agctacccatggccttgtcatca |
| Xenopus 4E-T |  |  |
| 4E-TForY28A  4E-TRevY28A | FLAG-MS2-4E-T | ggcagatctcatcacagtgcttcaaaggaggaacttttg  caaaagttcctcctttgaagcactgtgatgagatctgcc |
| 4E-TForA^28^AA  4E-TRevA^28^AA |  | ggcagatctcatcacagtgcttcaaaggaggaagctgcg  cgcagcttcctcctttgaagcactgtgatgagatctgcc |
| 4E-TForY53A  4E-TRevY53A |  | tcttgtttgcttgataaggctgacagtgatggtgtttg  caaacaccatcactgtcagccttatcaagcaaacaaga |
| 4E-TForA^53^AA  4E-TRevA^53^AA |  | aaggctgacagtgatggtgctgcggatcctgaaaaatggcat  atgccatttttcaggatccgcagcaccatcactgtcagcctt |
| Human 4E-T |  |  |
| 4E-TKpnIFor  4E-TXmaIRev | pEGFP-C1 | GCGTTAGGTACCATGGATAGGAGAAGTATGGGT  CTCAAGCCCGGGTCACTGTCGGTATTCCAATTC |
| 4E-T ForY30A  4E-T RevY30A | pEGFP-C1 | caaatgcccccatcgcgctacaaaagaagaactc  gagttcttcttttgtagcgcgatgggggcatttg |
| 4E-T ForA^30^AA  4E-T RevA^30^AA | pEGFP-C1 | GCTACAAAAGAAGAAGCCGCGGATATAAAAGAACTC  GAGTTCTTTTATATGCGCGGCTTCTTCTTTTGTAGC |
| 4E-T ForY55A  4E-T RevY55A | pEGFP-C1 | TGCCTTTCTGAAAAAGCTGACAGTGATGGTGTC  GACACCATCACTGTCAGCTTTTTCAGAAAGGCA |
| 4E-T ForA^55^AA  4E-T RevA^55^AA | pEGFP-C1 | GCTGACAGTGATGGTGCTGCGGACCCTGAGAAGTGG  CCACTTCTCAGGGTCCGCAGCACCATCACTGTCAGC |
| 4E-T ForLLAA  4E-T RevLLAA | pEGFP-C1 | TATACAAAAGAAGAAGCCGCGGATATAAAAGAACTC  GAGTTCTTTTATATGCGCGGCTTCTTCTTTTGTATA |
| 4E-TNdeIFor  4E-TBamHIRev | pGADT7 or  pGBKT7 | CGAGTGCATATGGATAGGAGAAGTATGGG  CGCTACGGATCCCTCACTGTCGGTATTCC |
| Human eIF4E2 |  |  |
| 4E2EcoRIFor  4E2XmaIRev | pEGFP-C1 | GCGTTAGAATTCTATGAACAACAAGTTCGACGCT  TGACCTCCCGGGCCTCATGGCACATTCAACCGCGG |
| 4E2ΔCBamHIFor  4E2ΔCXhoIRev | pcDNA3-HA | GCGTTAGGATCCATGAACAACAAGTTCGACGCT  GCGTTACTCGAGTCATTTGATGCTGTCGGTGTGAG |
| 4E2NdeIFor  4E2BamHIRev | pGADT7 or  pGBKT7 | CGAGTGCATATGAACAACAAGTTCGACGCT  CGCTACGGATCCCTCATGGCACATTCAACC |
|  |  |  |
